# Crystal structure of human persulfide dioxygenase: structural basis of ethylmalonic encephalopathy

**DOI:** 10.1093/hmg/ddv007

**Published:** 2015-01-16

**Authors:** Ilaria Pettinati, Jürgen Brem, Michael A. McDonough, Christopher J. Schofield

**Affiliations:** Chemistry Research Laboratory, University of Oxford, 12 Mansfield Road, Oxford OX1 3TA, UK

## Abstract

The ethylmalonic encephalopathy protein 1 (ETHE1) catalyses the oxygen-dependent oxidation of glutathione persulfide (GSSH) to give persulfite and glutathione. Mutations to the *hETHE1* gene compromise sulfide metabolism leading to the genetic disease ethylmalonic encephalopathy. hETHE1 is a mono-iron binding member of the metallo-β-lactamase (MBL) fold superfamily. We report crystallographic analysis of hETHE1 in complex with iron to 2.6 Å resolution. hETHE1 contains an αββα MBL-fold, which supports metal-binding by the side chains of an aspartate and two histidine residues; three water molecules complete octahedral coordination of the iron. The iron binding hETHE1 enzyme is related to the ‘classical’ di-zinc binding MBL hydrolases involved in antibiotic resistance, but has distinctive features. The histidine and aspartate residues involved in iron-binding in ETHE1, occupy similar positions to those observed across both the zinc 1 and zinc 2 binding sites in classical MBLs. The active site of hETHE1 is very similar to an ETHE1-like enzyme from *Arabidopsis thaliana* (60% sequence identity). A channel leading to the active site is sufficiently large to accommodate a GSSH substrate. Some of the observed hETHE1 clinical mutations cluster in the active site region. The structure will serve as a basis for detailed functional and mechanistic studies on ETHE1 and will be useful in the development of selective MBL inhibitors.

## Introduction

Ethylmalonic encephalopathy (EE; OMIM: 602473) is an inborn autosomal-recessive disorder that has severe gastrointestinal and neurological effects in infants (1–3). EE is caused by mutations to the gene (HGNC: 23287) encoding for the ethylmalonic encephalopathy protein 1 (ETHE1, also known as sulphur dioxygenase, SDO) (4–6) and correlates with increased cellular levels of hydrogen sulfide. Although hydrogen sulfide is highly toxic above low threshold levels, it is also proposed as a gaseous redox signalling molecule. Thus, abnormally increased hydrogen sulfide levels have the potential to result in highly pleiotropic and toxic effects consistent with the lethal phenotype observed in infants with EE (7). ETHE1 is proposed to play a role in other diseases, including acute myocardial infarction and cardiovascular disorders (8,9). A deficiency of hydrogen sulfide is proposed to be of pathophysiological relevance, arising perhaps as a consequence of cross-talk with nitric oxide or other reactive oxygen species involved in signalling (10). ETHE1 is reported to be localized to mitochondria where its activity is linked to electron transfer chain energy generation and is of central importance in hydrogen sulfide metabolism (11). ETHE1 is a non-heme iron-dependent oxygenase that catalyses the biochemically interesting oxidation of glutathione persulfide (GSSH) to give glutathione and persulfite (11) [Eq. (1)].

$$\text{GSSH + }{\text{O}}_{2}+{\text{H}}_{2}\text{O}\;\overset{\text{hETHE1}}{\to }\;{\text{GSH + SO}}_{3}^{2-}+2{\text{H}}^{+}.$$

**Figure 1.**
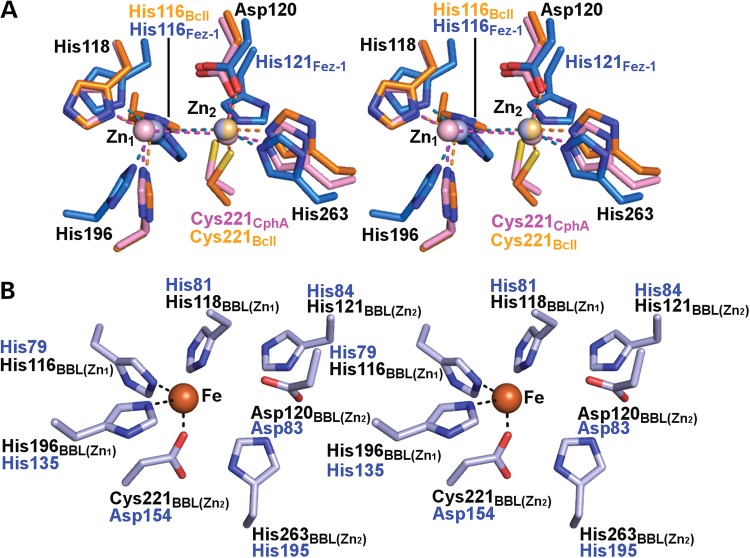
Comparison of hETHE1 active site with those of the Class B1, B2 and B3 prokaryotic MBLs. (**A**) Wall-eyed stereoviews of superimposed active site residues from the Class B1 MBL BcII from *Bacillus cereus* (PDB ID: 1BVT) (orange), the Class B2 MBL CphA from *Aeromonas hydrophila* (PDB ID: 3F9O) (pink) and the Class B3 MBL FEZ-1 from *Legionella gormanii* (PDB ID: 1K07) (blue). The standard BBL numbering system for MBLs is used (17). Residues present in all the three active sites are numbered in black, zinc ions are in light-orange (BcII), light-pink (CphA) and light-blue (FEZ-1). Note that the zinc-ligating residue His121 is only present in the Class B3 FEZ-1, whereas Cys221 is absent in the FEZ-1 B3 MBL compared with the Class B1 and B2 MBLs. The FEZ-1 active site residue composition is most similar to that of the hMBLs, despite the latter apparently displaying closer similarity with the Class B1 MBLs from an overall structural perspective (see Fig. 6). (**B**) Wall-eyed stereoview of the hETHE1 active site residues. The hETHE1 residue numbering is in blue and based on the enzyme sequence; BBL numbering is shown below in black. Note that in superimposition of hETHE1 with BcII (Fig. 6C), His79_ETHE1_ (His116_BBL_) does not correlate with His116_BBL_ of BcII, but with His118_BBL_ showing a different organization of conserved residues in their active sites. Note that the side chains of His84_ETHE1_ (His121_BBL_) and FEZ-1 His121_BBL_ are observed in different orientations in their respective active sites, probably because His121_BBL_ of FEZ-1 is involved in an additional metal binding (zinc 2 site), which is not observed in hETHE1.

Sequence analyses predict that hETHE1 is a member of the widely distributed metallo-β-lactamase (MBL)-fold family (5). MBL-fold proteins were first isolated from prokaryotes showing β-lactam antibiotic resistance (12); these classical MBLs are di-, or less commonly mono-, zinc-ion-dependent hydrolases; they act on almost all known β-lactam antibiotics, including carbapenems and are an increasing clinical concern (13–15). The first description of an MBL crystal structure (i.e. that of the *Bacillus cereus* MBL) revealed a new protein fold containing an alpha-beta-beta-alpha core fold and distinctive active site architecture (12). Subsequent studies revealed three classes of MBLs: B1, B2 and B3. The MBL enzymes are characterized by the presence of five highly, but not universally, conserved active site elements (motifs) distributed across the MBL-fold (16): motif 1, D84 (which is not directly involved in zinc binding); motif 2, H116-X-H118-X-D120; H121 is present in class B3 MBLs and some human MBL-fold enzymes (hMBLs); motif 3, H196; motif 4, C221; and motif 5, H263 (12,16,17). In classical di-zinc B1 MBLs these residues normally bind two neighbouring metal ions in the active site: zinc 1 is coordinated by the side chains of H116, H118 and H196. Zinc 2 is coordinated by the side chains of D120, C221 and H263 (Fig. 1). Subsequent work has revealed that the MBL-fold is extremely widespread and found in enzymes with a range of biological functions (18). In humans, MBL-fold enzymes have roles in detoxification [i.e. ETHE1 and hydroxyacylglutathione hydrolase (HAGH), also called glyoxalase II] (11,19), DNA repair (DNA cross-link repair 1A-B-C) (20) and RNA processing and maturation (cleavage and polyadenylation specific factor family members) (21,22). The diversity of biological roles observed for MBL-fold enzymes is apparently reflected by variations in their active site metallo-chemistry. At present, the available evidence suggests that the majority of MBL-fold enzymes employ one or two zinc ions for catalysis in hydrolytic reactions. However, many MBL-fold hydrolases, including the classical MBLs, are able to employ other metal ions [i.e. Mg(II), Ca(II), Co(II)] and/or mixtures of metals for catalysis (23,24). In this regard, ETHE1 is of particular interest because it employs a single non-heme iron ion to catalyse a reaction that is reminiscent of those catalysed by structurally unrelated non-heme iron-dependent oxygenases; in particular isopenicillin N synthase (IPNS), which is an unusual member of the iron and 2-oxoglutarate (2OG)-dependent oxygenase superfamily (25–27), and cysteine dioxygenase (28,29). These enzymes have related metal-coordination chemistry to the MBL-fold enzyme superfamily (30); here we report crystallographic studies on human ETHE1. The structural work provides insights into the effects of clinically observed *ETHE1* mutations (4–6), and, by comparison with the structure of an *Arabidopsis thaliana* ETHE1-like enzyme (31), shows active site features that distinguish the ETHE1s from other MBL-fold containing enzymes.

## Results

### Overall fold of hETHE1

Recombinant hETHE1 lacking its 20 residue N-terminal mitochondrial targeting sequence was produced in *Escherichia coli* and highly purified (≥95%) as determined by sodium dodecyl sulfate-polyacrylamide gel electrophoresis (SDS-PAGE) analysis (Supplementary Material, Fig. S1). hETHE1 was crystallized using the sitting drop vapour diffusion method. The final model (orthorhombic space group *P*2_1_2_1_2_1_) has two chains representing the dimer in the asymmetric unit.

**Figure 2.**
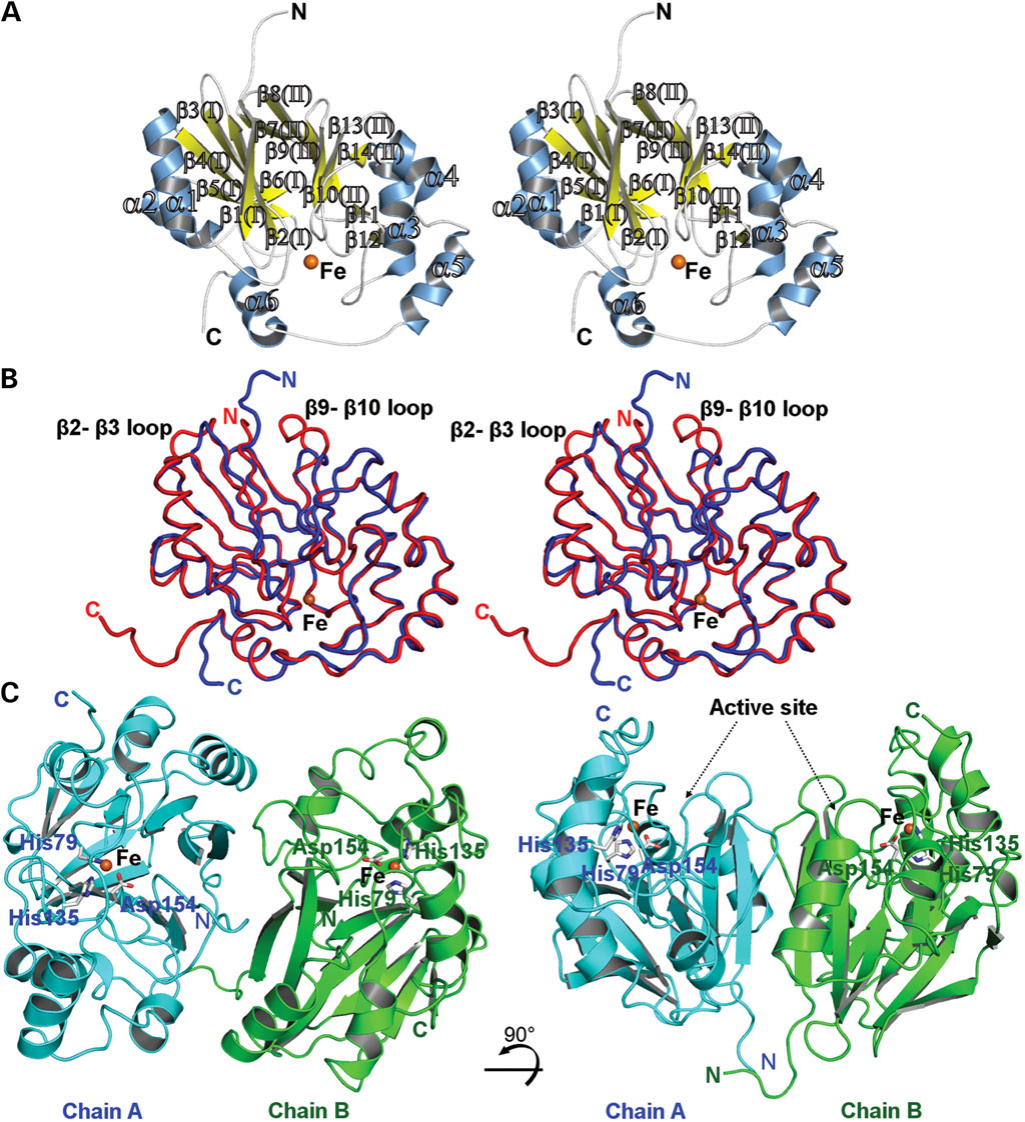
Views from the hETHE1 crystal structure. (**A**) Wall-eyed stereoview of hETHE1 showing secondary structure elements and the mono iron containing active site. Helices are blue, β-strands yellow and the iron is an orange sphere. (**B**) Wall-eyed stereoview of the superposition of hETHE1 (blue) and the *A. thaliana* ETHE1-like enzyme (red) (RMSD 1.43 Å over 230 Cα atoms). The structures reveal very similar overall folds except for small differences in the β2–β3 and β9–β10 loops. (**C**) The crystallographically observed hETHE1 dimer. Active sites for both chains exist on the same face of the dimer. Chains A and B are in cyan and green, respectively.

**Figure 3.**
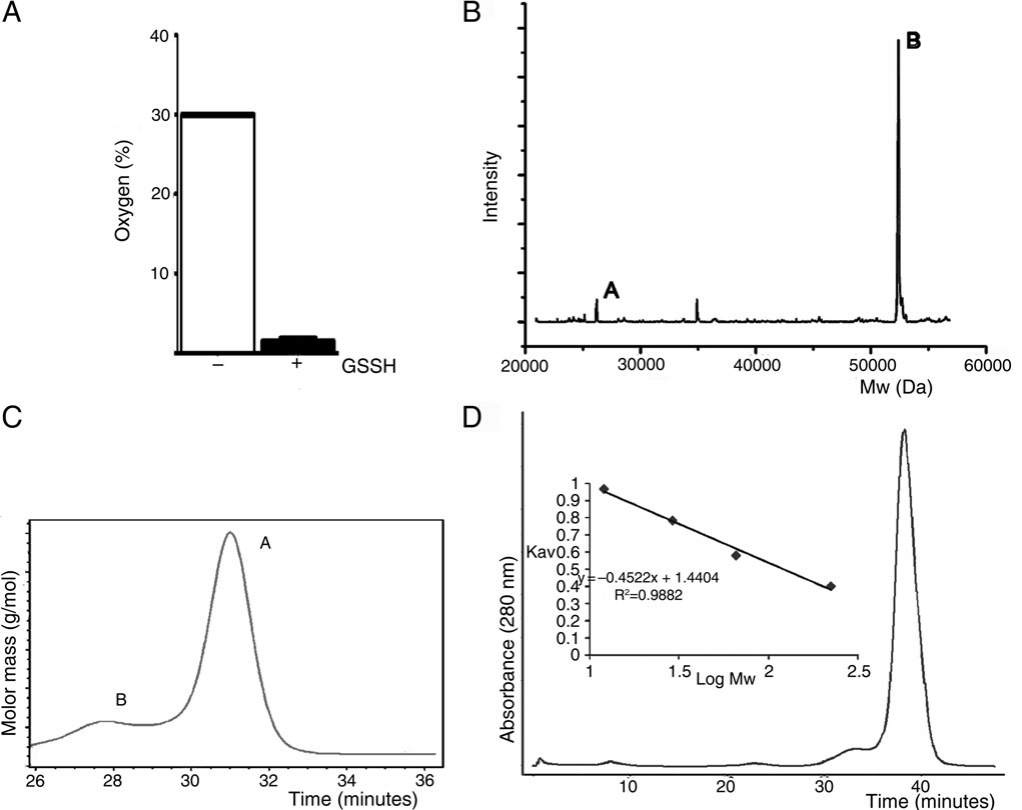
hETHE1 activity assay and oligomerization state. (**A**) Oxygen consumption activity assay. hETHE1 activity was measured as percentage of oxygen consumed in the presence of GSSH. Each sample was performed in triplicate. (**B**) Non-denaturing electrospray soft-ionization mass spectrometry deconvoluted spectrum of purified hETHE1 protein indicates that hETHE1 is primarily dimeric. Peak A (26 170 Da) represents monomer; peak B (52370 Da) the dimer. In both monomeric and dimeric states one iron ion (+56 Da) is bound to each protomer while only in the dimer a +30 Da was observed possibly due to the oxidation state of Cys247 (see the main text and Supplementary Material, Fig. S5) (conditions: 15 µm of hETHE1 in 15 mm ammonium acetate buffer (pH 7.5); cone voltage for the acquisition of the spectra was 80 V). (**C**) MALS analysis of hETHE1 protein after purification. Peak A (∼51 560 Da) represents monomer; peak B (∼99 190 Da) the tetramer. MALS experiments were carried out by the Biophysical Services of the Biochemistry Department of Oxford University. (**D**) The molecular mass of hETHE1 in solution was estimated using a Sephadex G250 gel filtration column calibrated with protein standards [beta-amylase (223 kDa), albumin (66 kDa), carbonic anhydrase (29 kDa) and cytochrome C (12 kDa)].

The hETHE1 structure reveals an αββα MBL-type fold with two central mixed β-sheets, each containing six strands, surrounded on both sides by helices (Fig. 2A and Supplementary Material, Fig. S2). In β-sheet I, β-strands 1–3 are aligned anti-parallel, with β-strands 3–6 being parallel. In β-sheet II, β-strands 7, 8, 9 and 10 are anti-parallel; β-strands 10 and 13 are parallel and β-strands 13 and 14 are anti-parallel (Supplementary Material, Fig. S2A). Secondary structural elements include: 2 β-sheets, 2 βαβα units, 8 β-hairpins, 6 β-bulges, 14 β-strands, 6 helices, 2 helix–helix interactions and 26 β-turns. Superimposition of the hETHE1 and *A. thaliana* ETHE1-like (PDB ID: 2GCU) structures reveals high overall fold similarity [root-mean-square deviation (RMSD) 1.43 Å over 230 Cα atoms] between the two proteins (Fig. 2B). Structure-based topology diagrams show conserved structural organization with the exception of the addition of the β11–β12 hairpin in the region linking β10 and β13 of the core fold of hETHE1 compared with the *A. thaliana* ETHE1 (Supplementary Material, Figs S2A and B). Topology comparisons of hETHE1 with human glyoxalase II (HAGH) and a ‘classical’ metallo-β-lactamase II (BcII) from *B. cereus* (Supplementary Material, Fig. S2C and D) reveal much more substantial differences in the organization of secondary structure elements. hETHE1 has been assigned as a member of the glyoxalase II family on the basis of sequence alignments (31); however, consistent with the differences in their overall folds (19), hETHE1 does not display glyoxalase II activity, when assayed using the most common glyoxalase II substrate, *S*-(D)-lactoylglutathione, under standard conditions (11). Enzyme-dependent oxygen consumption activity in the presence of GSSH was observed using the oxygen consumption assay as previously reported (11) (Fig. 3A). Moreover, ETHE1 does not display β-lactamase activity either using a chromogenic cephalosporin (nitrocefin), a penicillin (penicillin G) or a carbapenem (meropenem) as substrates under our standard β-lactamase assay conditions (32).

As reported for the *A. thaliana* protein (33), hETHE1 is predominantly a dimer in solution. Non-denaturing mass spectrometric and multi-angle laser light scattering (MALS) analyses indicate that hETHE1 is predominantly dimeric with some monomer also being observed (dimer to monomer ratio ∼10:1); quantitative gel filtration analysis indicated a mass for hETHE1 intermediate between monomeric (26 116 Da) and dimeric (52 232 Da) forms in solution (Fig. 3B–D). The crystal packing of the human ETHE1 is similar to that reported for the *A. thaliana* ETHE1-like structure (31), with both hETHE1 and *A. thaliana* ETHE1-like crystallizing as dimers. Interactions at the crystallographically observed hETHE1 dimer interface were analysed by PISA (34) to investigate their potential for functional relevance. The total buried surface area at the interface is 1950 Å^2^ and the calculated free energy of binding (Δ*G*) for the dimer is about −37 kcal/mol. The calculated complex formation significance score (CSS) value of 0.351 output from PISA is consistent with the observed dimer formation in solution (Fig. 2C). Notably, conserved interactions are present at the dimer interface of both human and *A. thaliana* ETHE1-like even though the proteins were crystallized under very different conditions.

### hETHE1 active site

**Figure 4.**
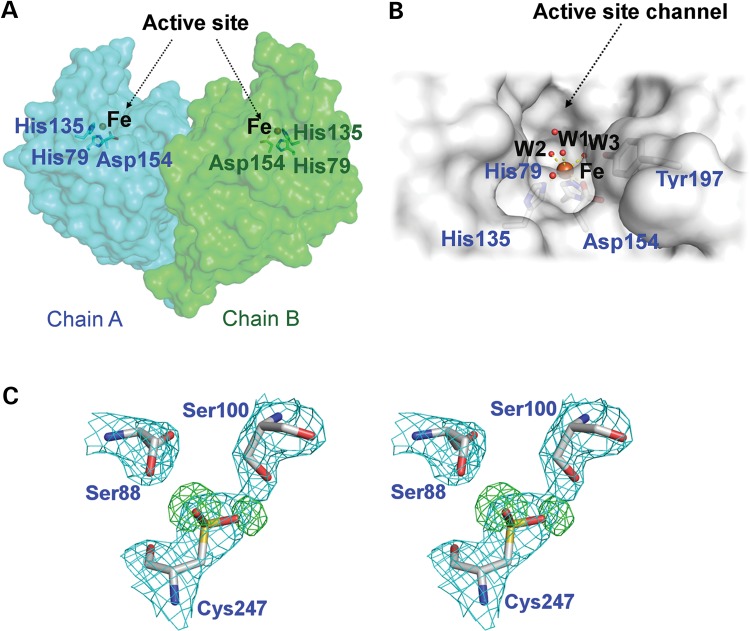
hETHE1 surface analysis. (**A**) Surface representation of the crystallographically observed hETHE1 dimer (chain A cyan, chain B green). Metal binding residues shown as sticks, iron ion shown as orange sphere. (**B**) Surface representation of the active site groove showing the side chain of Tyr197 directed towards the metal (6.8 Å). (**C**) The side chain of Cys247 was refined as a sulfinic acid (CSD247) (i.e. RSO_2_H) as observed in the experimental electron density (3.0 *σ* mFo-DFc OMIT, omitting oxygen atoms; green mesh). The two serine residues (Ser 88 and Ser 100) are only weakly conserved whilst Cys247 is conserved in most predicted ETHE enzymes as shown in Figure 7.

**Figure 5.**
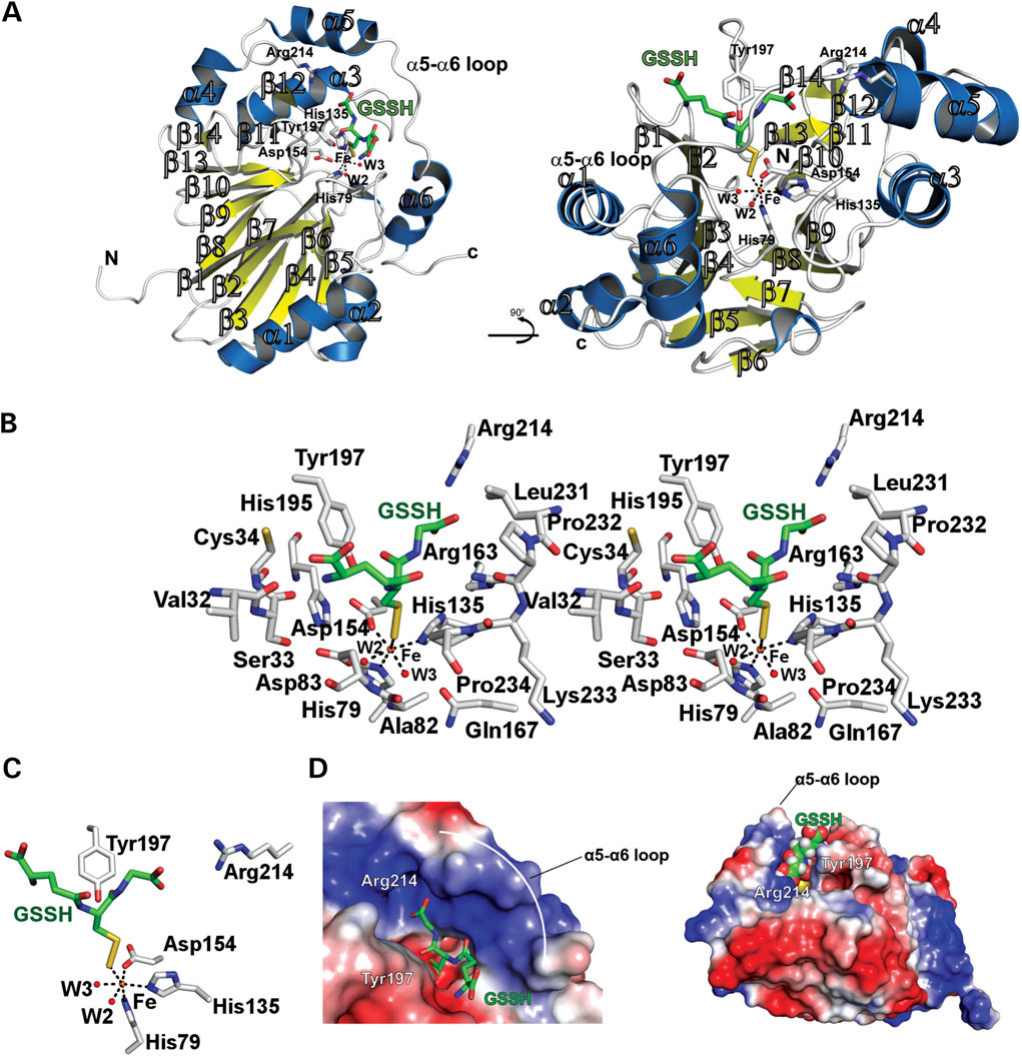
Docking of GSSH in hETHE1 active site. GSSH was manually docked into the active site groove using the shape of the groove and an electrostatic surface potential map as a guide. The GSSH thiol was restrained near the metal ion and the glycyl carboxylate of GSSH was orientated towards the positively charged end of the pocket so that the primary amine on the opposite end of the chain faced the negatively charged surface. The ligand is surrounded by the α5–α6 loop on one side of the groove and by Tyr197 on the other side. All manual docking was performed using Pymol. (**A**) Ribbons representation of GSSH manually docked into the ETHE1 active site. (**B**) Wall-eyed stereoview of the hETHE1 active site and surrounding residues possibly participating in substrate binding and/or stabilization. (**C**) Enlarged view of GSSH docked in the hETHE1 active site. (**D**) Surface representation of GSSH docked in hETHE1 active site groove. Note the possible substrate interaction with Tyr197 and Arg214.

The active site of each hETHE1 protomer in the asymmetric unit contains a single iron ion (Fig. 2C). Analysis of the enzyme surface reveals a channel, comprised of residues 163–166 and 226–232, leading to the active site that is sufficient to accommodate a substrate molecule of GSSH (Fig. 4A and B); this channel is apparently conserved in the *A. thaliana* ETHE1. Residues making up the channel may also act in the stabilization of intermediates and/or product release. Manual docking of GSSH into the hETHE1 active site (Fig. 5) is consistent with the proposed mechanism of catalysis involving an Fe-SSG intermediate (Supplementary Material, Fig. S3).

**Figure 6.**
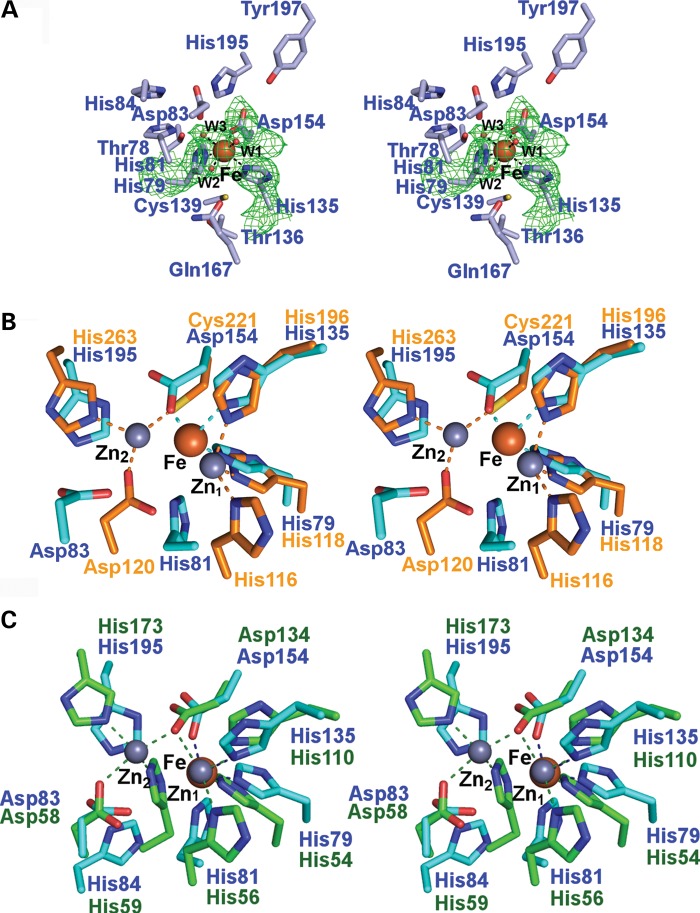
hETHE1 active site and comparison of the hETHE1 crystal structure with other MBL-fold enzymes. (**A**) Wall-eyed stereoview of the iron binding and active site residues of hETHE1 with representative electron density (3.0 *σ* mFo-DFc OMIT; green mesh) for side chains of His79 (Nϵ2 to Fe: 2.3 Å), His135 (Nϵ2 to Fe: 2.22 Å), Asp 154 (Oδ2 to Fe: 2.05 Å) and the three water molecules (red spheres) which coordinate (black dashed lines) to the iron (orange sphere). (**B**) Wall-eyed stereoview of superimposed active site residues from hETHE1 (cyan) and BcII from *Bacillus cereus* (PDB ID: 1BVT) (orange). The zinc and iron ions are in grey and dark red, respectively. The zinc ions in the Zn_1_ and Zn_2_ sites are labelled (17). There is relatively strong conservation in iron-binding residues by ETHE1 and at the Zn_1_ site of glyoxalase II; although the Zn_2_ binding site residues are conserved in hETHE1, they do not bind the iron ion. (**C**) Wall-eyed stereoview of the superimposed active site residues from hETHE1 (cyan) and human glyoxalase II (PDB ID: 1QH3/5) (green). *Note*. There are more differences between hETHE1 and BcII than between hETHE1 and glyoxalase II.

Interestingly, three of five characteristic MBL family metal-binding motif residues (35), His79, His135 and Asp154 (corresponding to His118, His196, and Cys221, BBL numbering system), act as iron coordinating residues in hETHE1 (Fig. 6A). Three water molecules complete the metal coordination. Residues His81_ETHE1_ (His118_BBL_), Asp83_ETHE1_ (Asp120_BBL_) and His195_ETHE1_ (His263_BBL_) are present in the hETHE1 active site, but are not involved in metal coordination as observed in the current structural data. The position of Asp83_ETHE1_ and His195_ETHE1_ somewhat resembles the zinc 2 organization of the classical MBLs (12) (Fig. 6B). However, the side chain carboxylate of Asp52_ETHE1_ (Asp84_BBL_) is ∼6 Å from the iron ion.

Comparison of the hETHE1 and *A. thaliana* ETHE1 structures reveals similar iron binding with near identical orientations and metal distances between conserved residues (Fig. 2B). Comparison of the active sites of hETHE1 and human glyoxalase II (HAGH) (which binds two zinc ions) shows that they display partial similarity in zinc 1 coordinating residues: HAGH uses His54_GII_, His110_GII_ and Asp134_GII_ to coordinate zinc and hETHE1 uses His79_ETHE1_, His135_ETHE1_ and Asp154_ETHE1_ to coordinate iron. The major difference between the hETHE1 and hHAGH active sites is the number of bound metals, with zinc 2 of hHAGH interacting with conserved MBL motif residues, Asp58_GII_, His59_GII_ and His173_GII_, which correspond to Asp83_ETHE1_, His84_ETHE1_ and His195_ETHE1_ of ETHE1 (Asp120_BBL_, class B3 His121_BBL_ and His263_BBL_). The similarity of residues used for zinc 2 binding in hHAGH compared with those in the hETHE1 active site may explain why hETHE1 has been previously identified as a close relative of the glyoxalase II family (19) (Fig. 6C).

**Figure 7.**
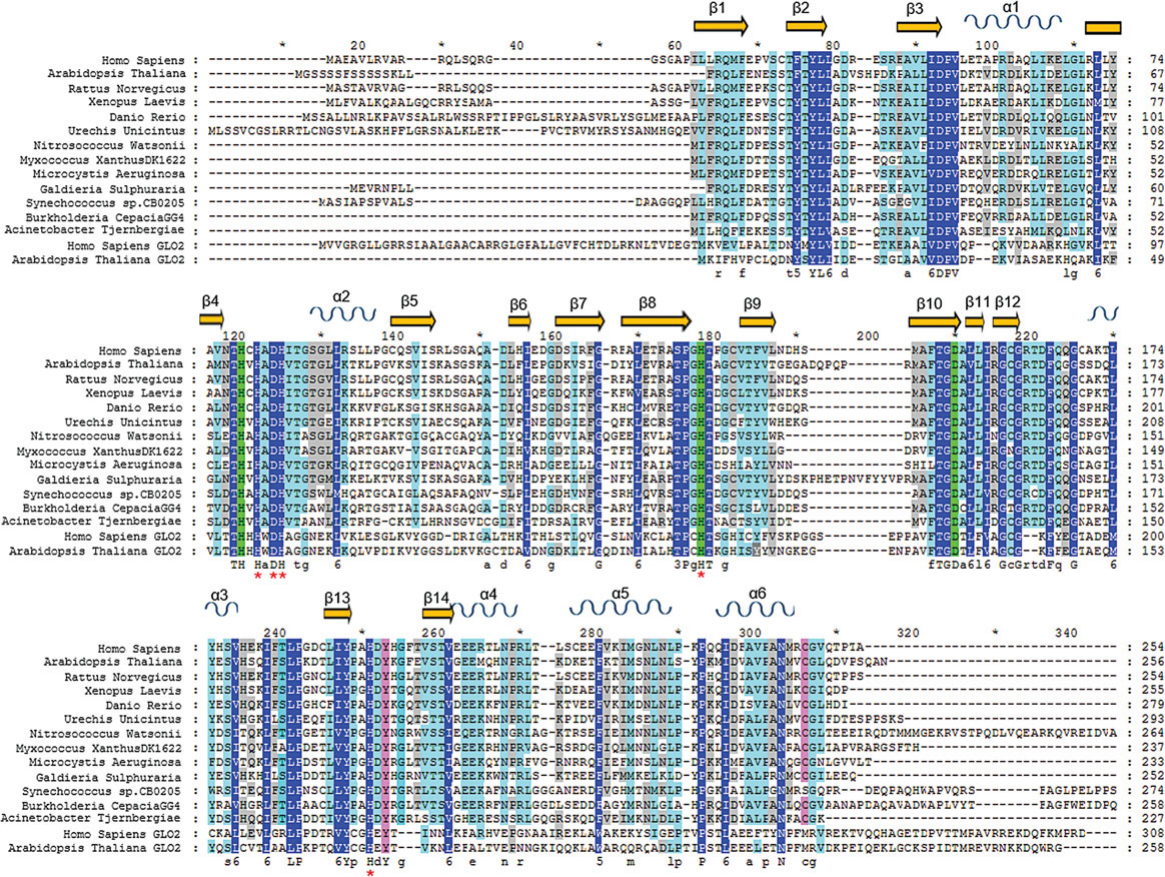
Multiple sequence alignment of ETHE1 from different organisms. COBALT BLAST (36) was used to align sequences identified in a BLAST search (37). The hETHE1 sequence was used as the query and the secondary structure elements are derived from the human ETHE1 structure (PDB ID 4CHL). Secondary structure elements were inserted using the ESPRIT 3 tool (http://espript.ibcp.fr) (38). β-Sheets are shown as yellow arrows, and α-helices as blue sinusoidal waves. Residues are coloured based on conservation: dark blue represents the highest conservation grade, light blue the second highest, grey the third highest and no colour the least conserved. The MBL-fold proteins glyoxalase II from *Homo sapiens* and *Arabidopsis thaliana* were added to the multiple sequence alignment. The three iron-binding residues (His79, His135 and Asp154) are highlighted in green; Tyr197 and Cys247 are in pink. Red asterisk (*) indicates a site 2 Zn binding residue of Glyoxalase II (note that Asp134 binds both zinc ions in the Glyoxalase II active site).

Superimposition of the hETHE1 structure with that of the prototypical bacterial MBL class B1 enzyme, BcII, also reveals a similar active site. The BcII MBL has two zinc ions in its active site with zinc 1 coordinated by three histidines (His116_BBL_, His118_BBL_ and His196_BBL_), and zinc 2 coordinated by Asp120_BBL_, Cys221_BBL_ and His263_BBL_ (Fig. 6B). Sequence comparison reveals that Cys221_BBL_ is apparently not present in the metal-binding motifs of hMBLs (Fig. 7).

Two residues of interest in the hETHE1 structure are Cys247 and Tyr197. hETHE1 appears to be modified as shown by non-denaturing mass spectrometry experiments by the addition of two oxygen atoms and Cys247 was refined as a cysteinyl sulfinic acid (RSO_2_H) (Protein Data Bank acronym: CSD) (Figs 3B and 4C). We considered that the observed oxidation state of Cys247 may be an artefact derived from protein preparation. Interestingly, however, crystallographic analysis has revealed that the equivalent cysteine (Cys246) in the *A. thaliana* ETHE1-like structure is also observed to be doubly oxidized supporting the potential for a function for this residue. The possibility of autocatalytic oxidation of Cys247 in ETHE1 is well precedented in metallo-enzymes including nitrile hydratase, where two of three active site clustered cysteine residues are post-translationally oxidized to sulfinic acid and sulfenic acid, respectively, modifications that are essential for catalytic activity (39,40). The oxidation of Cys247 is also notable because the hETHE1 reaction involves oxidation of a thiol to give persulfite [Eq. (1)]. It is thus possible that oxidation of Cys247 is either of catalytic relevance or represents a non-productive/damaging protein oxidation, as is well precedented in other oxygenases and metallo-proteins (41) including haemoglobin (42). Tyr197 is highly conserved in ETHE1 across different organisms including *A. thaliana* (31), but it is not apparently conserved among prokaryotic MBLs or hMBL-fold enzymes with the exception of HAGH (19) and paroxysmal nonkinesigenic dyskinesia protein (PNKD) (43). Tyr197 is positioned in the active site with its side chain hydroxyl directed towards the active site metal (Figs 4B and 5). Given the proposed role of electron transfer in the catalytic mechanism of ETHE1 as shown in Supplementary Material, Figure S3, it is possible that the phenolic group of Tyr197 has an active role in catalysis, as proposed for some other non-heme iron-dependent oxygenases, e.g. carbapenem synthase (44,45).

### Mapping clinically observed EE mutations on the hETHE1 structure

**Figure 8.**
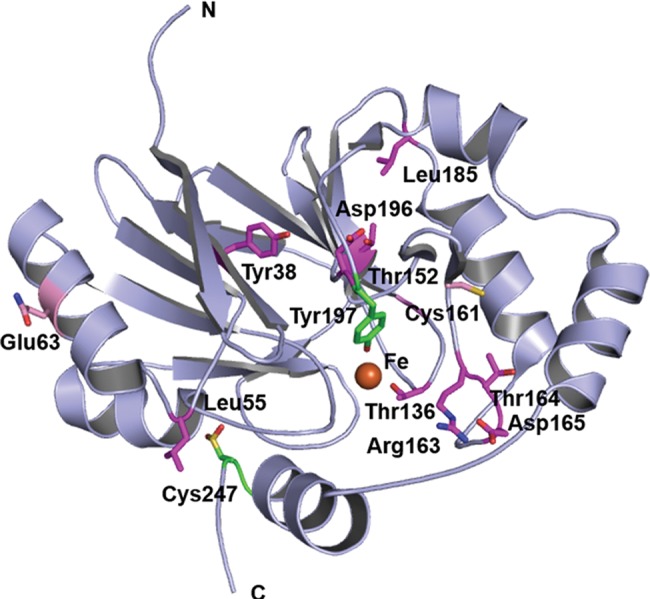
Clinically observed mutations in *hETHE1*. Clinically observed substitutions mapped onto the hETHE1 crystal structure. Position of hETHE1 substituted residues (sticks) mapped onto the crystal structure (PDB ID: CHL4). Structure-based sequence analysis using ConSurf reveals that substitutions occur at medium (pink) or highly conserved (magenta) residues (47,48). Tyr197 and the oxidized Cys247 are in green.

Mutations have been identified in the *h*ETHE1 gene of individuals with EE (Table 1) (6). We used the hETHE1 structure to map 13 missense-mutations resulting in EE disease (Fig. 8; substituted residues in pink or magenta). Interestingly, the Asp196 substitution is positioned immediately before Tyr197 and is reported to lead to decreased substrate affinity (6,11), supporting a proposed role for Tyr197 in substrate binding/catalysis. Moreover, with the exception of Gln12, all EE disease correlating substitutions occur at positions of conserved (Glu63, Cys161), or highly conserved (Tyr38, Leu55, Thr136, Thr152, Arg163, Thr164, Asp165, Leu185 and Asp196), residues, reflecting their likely importance in protein function and maintenance of overall structure (Fig. 8). Interestingly, substitutions resulting in EE disease have been observed at residues lining the proposed substrate binding channel, Arg163, Tyr164 and Asp165 (5,6). The structural information supports the recent discovery that mutations occurring at Arg163 are able to alter hETHE1 stability and the active site metal chemistry (49). Some of the substitutions involve residues preceding or following the active site iron-binding residues, in particular, variants of Thr136, Thr152 and Asp196 (i.e. adjacent or close to the metal coordinating residues, His135, Asp154 and His195, respectively). Substitutions at these likely crucial positions in the structure could lead to instability and/or conformational changes to the active site (11) and may help to explain how a single point mutation results in a catalytically compromised hETHE1.

**Table 1.** Clinically observed mutations in hETHE1 that correlate with EE

Residue number	Substitution		Reference/reported substitution effects
	From	To	
12	Q	X	(4)
38	Y	C	Reduced protein stability (5)
55	P	L	Reduced protein production (6)
63	Q	X	(4)
136	T	A/G	(5,6)
152	T	I	Reduced protein stability, iron content, enzyme activity (6,11)
161	C	Y	(4)
163	R	Q/W/G	Reduced protein stability, reduction potential of iron, enzyme activity (46)
164	T	K	Reduced protein stability (5,6)
165	D	G	(5)
185	L	R	(6)
196	D	N	Reduced protein stability/substrate affinity (6,11)

Reported clinical mutations for hETHE1 that result in EE (4–6,11). Where reported, the effects of the mutation at the protein level are given. Substitutions indicated with X represent nonsense mutations that encode for truncated versions of hETHE1. Protein stability was measured based on the levels of ETHE1-protein-specific cross-reacting material (CRM) present in fibroblasts as detected by western blotting (5).

## Discussion

The structural analyses clearly demonstrate that hETHE1 is an MBL-type fold as predicted by sequences analysis (31). The results also reveal that there are clearly specific differences in the active site metal binding of hETHE1 compared with both other eukaryotic MBL-fold enzymes (e.g. glyoxalase II) (19) and the classical bacterial MBLs (12); however, the degree of conservation is also striking. It is particularly interesting that residues involved in ‘classical’ zinc 1 and zinc 2 MBL binding are substantially (but not completely) conserved all the way through to the single-iron ion binding eukaryotic MBL-fold enzyme ETHE1 (5,12). The classical di-zinc MBLs can operate with metal ions other than zinc and use either one or two metal ions (50). Thus, it seems that the apparent conservation of possible ‘second’ metal-binding site residues in ETHE1 (and potentially other MBL-fold enzymes) is of functional significance. One possibility is that the activity of ETHE1 is regulated by binding of different metals, or that a di-metallic ETHE1 would have an alternative catalytic activity. It is also interesting that conserved residues in the active site of hMBLs are more similar to those present in the Class B3 bacterial MBLs compared with those of Class B1 or B2 based on sequence alignment, despite the observation that from a structural fold perspective hMBLs display higher similarity with the Class B1 and B2 bacterial MBLs in their active site architecture (Fig. 1) (12,34). It has been proposed that hMBLs evolved from an ancestor of the class B1–B2 prokaryotic MBLs based on their structural similarity (35,51), but it is also possible that eukaryotic MBL-fold proteins evolved from Class B3 bacterial MBLs, or from an ancient ancestor common to all three bacterial MBL classes.

The elucidation of the hETHE1 structure also reveals striking similarity of iron binding by enzymes of completely different overall fold, e.g. the alpha-beta-beta-alpha MBL-fold and the double-stranded beta-helix fold metal binding superfamily (12,26). Perhaps the most striking similarity is between ETHE1 and IPNS, both of which employ a facial triad of iron-binding residues and whose mechanisms both employ Fe-S–peptide interactions, through Fe-SCH_2_R in the case of IPNS (25) and Fe-S-SCH_2_R in the case of ETHE1 (11). Indeed the structural similarity between MBLs and IPNS was first observed on the determination of the BcII MBL structure in 1995 (12). The determination of the hETHE1 structure may enable the development of a detailed mechanistic understanding for ETHE1, as has been the case for crystallographic and spectroscopic studies on IPNS (25,52) and cysteine dioxygenase (28,29). The hETHE1 crystal structure will serve as a basis for detailed mechanistic studies and for insights into the basis of clinically observed substitutions causing impaired hydrogen sulfide metabolism. In this regard the observation that a cysteine residue is apparently oxidized to a sulfinic acid in hETHE1 and the ETHE1-like in *A. thaliana* is of interest. In the longer term it is hoped that structural insights may help to enable treatment of EE; although the development of such treatments are probably a long way off, it is notable that at least some of the clinically observed substitutions are in the putative substrate binding site, suggesting that impaired substrate binding may be an issue. Multiple metallo-enzymes are already used or are being pursued as pharmaceutical targets, with very substantial medicinal chemistry efforts being used to develop selective inhibitors (16,53,54). Some of these enzymes employ very similar metal coordination arrangement as observed for hETHE1. Such enzymes include MBLs themselves (46) in efforts to combat antibiotic resistance and, e.g. the iron and 2OG oxygenases, e.g. the hypoxia inducible-factor prolyl-hydroxylases and histone demethylases (52). Most of the inhibitors of these enzymes, and all of those in clinical trials, are active site binding iron chelators, which may well bind to MBL-fold iron or zinc ion utilizing enzymes (55,56). Since inhibitors of hETHE1 will likely have toxic side effects, the structural work will help to enable the development of selective metallo-enzyme inhibitors.

## Materials and Methods

### Protein production and purification

cDNA encoding for hETHE1 (Uniprot ID: O95571), lacking its 20 residue N-terminal mitochondrial signal sequence, was inserted into a pCOLD I vector (*Addgene*) to encode for hETHE1 with an N-terminal hexa-histidine tag including an N-terminal 3C human rhinovirus (HRV3C) protease cleavage site. Recombinant hETHE1 protein was produced in *E. coli* BL21 (DE3) cells. Cells were grown in 2TY growth media with 50 µg/ml ampicillin at 37°C to mid-exponential phase (OD 600 = 0.6–0.8). hETHE1 production was induced, by addition of 0.5 mm isopropyl β-d-1-thiogalactopyranoside (IPTG) and supplemented with 50 µm iron sulfate while incubating at 15°C. Cells were harvested by centrifugation (6500 *g*, 8 min; centrifuge Avanti, JA-10 rotor; Beckman Coulter, Inc.), after 18 h and frozen in liquid nitrogen. About 30 g of cell pellet were added to 100 ml of lysis buffer (20 mm 4-(2-hydroxyethyl)-1-piperazineethanesulfonic acid, 500 mm sodium chloride and 5 mm imidazole; the pH was then adjusted to 7.5), lysed by sonication followed by centrifugation (20 000 *g*, 20 min; centrifuge Avanti; JLA 16.25 rotor; Beckman Coulter, Inc.). hETHE1 was purified by loading the supernatant containing hETHE1 onto a 5 ml Ni-affinity column (Supplementary Material, Fig. S1A). The equilibration buffer was the same as the lysis buffer. The elution buffer additionally contained 500 mm imidazole used to form increasing imidazole concentration steps (from 5 to 500 mm) to elute His-tagged protein. hETHE1 (2 ml) was then loaded onto a S200 (300 ml) gel filtration column in 20 mm 4-(2-hydroxyethyl)-1-piperazineethanesulfonic acid, 500 mm sodium chloride, pH adjusted to 7.5 running buffer. Fractions containing protein were analysed by SDS-PAGE using 12% bis-acrylamide gels (Supplementary Material, Fig. S1B). The 6X-His tag was cleaved by addition of HRV3C protease and incubation overnight at 4°C and passed over a Ni-affinity column to remove the cleaved tag from the sample (Supplementary Material, Fig. S1C). Purified hETHE1 (∼26 kDa) was buffer exchanged into 50 mm 4-(2-hydroxyethyl)-1-piperazineethanesulfonic acid, pH 7.5, 100 mm sodium chloride and concentrated to 10 mg/ml using a Centricon concentrator (10 k MW cutoff) centrifuged at 1200*g* (Centrifuge Allegra X-30, SX4400 rotor; Beckman Coulter, Inc.), until the desired volume was achieved.

### Glyoxalase II activity assay

The glyoxalase activity of hETHE1 was tested using *S*-D-lactoylglutathione as described (19). The reaction was performed in a final volume of 200 µl, and monitored by absorbance detection using a plate reader (BMG LABTECH PHERAstar FS) in 96 well plates. The final assay mixture contained 130 µg of enzyme, 1 mm *S*-D-lactoylglutathione and 200 µm 5,5-dithiobis-(2-nitrobenzoate) (DTNB) dye (extinction coefficient at 37°C: 13 600 M^−1^ cm^−1^) in 100 mm 3-(*N*-morpholino)propanesulfonic acid (MOPS), pH 7.2 buffer. Real-time glutathione formation in presence of 5-thio-2-nitrobenzoic acid was monitored at 412 nm and at 37°C.

### Oxygen consumption assay

Persulfide substrate was prepared as previously described (11). Briefly, GSSH was prepared by reacting NaHS and oxidized glutathione (GSSG) under anaerobic conditions. An oxygen depleted solution of 20 mm GSSG in 100 mm sodium phosphate, pH 7.4 [100 mm sodium phosphate buffer was obtained by mixing 3.1 g of NaH_2_PO_4_ and 10.9 g of Na_2_HPO_4_ (anhydrous) in distilled water to give a final volume of 1 l] was mixed with an excess of NaHS. The reaction was sealed and incubated at 37°C for 30 min. hETHE1 activity was measured in terms of oxygen consumption during the substrate (GSSH) catalysis. A FOXY AL-300 probe and an Ocean Optics USB2000/USB2000-LS-450 spectrophotometer were used for oxygen detection. Samples were prepared as follows: 1 µg of hETHE1 in 25 mm HEPES, pH 7.4, 200 mm NaCl was loaded into a sealed 2 ml vial in oxygen saturated 100 mm phosphate buffer, pH 7.4. GSSH was added to a final concentration of 1 mm immediately before the measurement. The amount of oxygen is expressed as the percentage of saturation. Control samples were carried out in the absence of GSSH.

### Crystallization and structure determination

hETHE1 crystallization was performed using the sitting drop vapour diffusion method in *Art Robbins* 96 wells—3 subwell Intelliplates^®^ and 300 nl size drops were obtained by adding the following ratios of protein solution and reservoir buffer: 200:100,100:100, 100:200 nl to the individual subwells. The truncated hETHE1 crystallized in ∼2 weeks using the following conditions: SaltRX condition 91, 0.1 m Tris–HCl, pH 8.5, 0.5 m potassium thiocyanate (protein to reservoir ratio 2:1 and 1:1) (Hampton Research, Aliso Viejo, CA). The resulting rhombohedron-shaped crystals (∼50 × 50 µm) were cryo-protected in well solution diluted to 25% glycerol (v/v) for 30 s, then harvested using nylon loops followed by cryo-cooling and storage in liquid nitrogen. Data were collected on a single crystal at 100 K at the Diamond Light Source synchrotron (beamline I04) to 2.6 Å resolution. Data were autoprocessed at the beamline using XDS (57) and CCP4-SCALA (58) in XIA2 (59). The hETHE1 structure was solved by molecular replacement (MR) using the PHASER subroutine within PHENIX (60–62) with the *A. thaliana* ETHE1-like structure (PDB ID: 2GCU) (31) as a search model. Refinement was carried out by iterative rounds of model building using Coot (63) and maximum likelihood restrained refinement using PHENIX (64). Data collection, processing and structure refinement statistics are given in Table 2.

**Table 2.** Crystallographic data and refinement statistics PDB ID 4CHL

Data set	Native hETHE1
Data collection	
Source	Diamond light source I04 beamline
Wavelength (Å)	1.07188
Resolution range (Å)	63.631–2.61 (2.68–2.61 Å)^d^
Space group	*P*2_1_2_1_2_1_
Unit cell parameters	
*a*, *b*, *c* (Å)	73.94, 124.93, 63.09
*α*, *β*, *γ* (°)	90.00, 90.00, 90.00
Reflections (unique)	18351
Completeness (%)	99.7 (99.2)^d^
Redundancy	6.5 (6.8)^d^
R*merge*^a^	0.12 (0.889)^d^
<*I*/*σ* (*I*)>	13.7 (1.9)^d^
Refinement	
R*cryst*^b^/R*free*^c^	0.178/0.223
Anisotropy	0.754
RMSD	
Bonds (Å)	0.01
Angles (°)	1.124
Average B, protein atoms (Å^2^)	49.0
Ramachandran plot (%)	
Most favoured geometry	99.1
Additionally allowed	0.9
Outliers	0

^a^R*merge* = *Σ_h_Σ_l_*|*I_hl_* − <*I_h_*>|/*Σ_h_Σ_l_*<*I_h_*>, where *I_hl_* is the *l*th observation of reflection *h*, and <*I_h_*> is the mean intensity of that reflection.

^b^R*cryst* = *Σ*||*F*_obs_ | − |*F*_calc_||/|*F*_obs_|.

^c^R*free* is calculated in the same way as R*cryst* but using a test set containing 5.13% of the data, which were excluded from the refinement calculation.

^d^Values for highest resolution shell.

## Accession Number

Coordinates and structure factors have been deposited in the Protein Data Bank with accession number 4CHL.

## Supplementary Material

Supplementary Material is available at *HMG* online.

## Funding

Medical Research Council (MRC)/Canadian grant G1100135 (I.P.) and the Biotechnology and Biological Sciences Research Council (BBSRC; C.J.S). Cancer Research UK (CRUK) is acknowledged for supporting J.B. and C.J.S. Funding to pay the Open Access publication charges for this article was provided by the Oxford's RCUK Open Access Block Grant.

## Supplementary Material

Supplementary Data
