# Supplementary material for: Crystal structure of human persulfide dioxygenase: structural basis of ethylmalonic encephalopathy
Source: Hum Mol Genet. 2015 Jan 16;24(9):2458–69. doi: 10.1093/hmg/ddv007 (PMC4383860; doi:10.1093/hmg/ddv007)
Supplement: Supplementary Data [file supp_24_9_2458__index.html]

Crystal Structure of Human Persulfide Dioxygenase: Structural Basis of Ethylmalonic Encephalopathy — Crystal structure of human persulfide dioxygenase: structural basis of ethylmalonic encephalopathy — Crystal structure of human persulfide dioxygenase: structural basis of ethylmalonic encephalopathy — Crystal structure of human persulfide dioxygenase: structural basis of ethylmalonic encephalopathy — Supplementary Data 

# Crystal structure of human persulfide dioxygenase: structural basis of ethylmalonic encephalopathy

## Supplementary Data

Supplementary Data

**Files in this Data Supplement:**

- Supplementary Data - Pdf file
