# Supplementary material for: Crystal structure of human persulfide dioxygenase: structural basis of ethylmalonic encephalopathy
Source: Hum Mol Genet. 2015 Jan 16;24(9):2458–69. doi: 10.1093/hmg/ddv007 (PMC4383860; doi:10.1093/hmg/ddv007)
Supplement: Supplementary Data [file supp_ddv007_ddv007supp.pdf]

Supplemental Information

Crystal Structure of Human Persulfide Dioxygenase: Structural Basis of Ethylmalonic Encephalopathy

Ilaria Pettinati[1], Jürgen Brem[1], Michael A. McDonough[1*], and Christopher J. Schofield[1*]

[1]From the Department of Chemistry, University of Oxford,12 Mansfield Road, Oxford, OX1 3TA, United Kingdom.

*To whom correspondence should be addressed: Dr. Michael A. McDonough and Prof. Christopher J. Schofield, the Department of Chemistry, University of Oxford,12 Mansfield Road, Oxford, OX1 3TA, UK, Tel: (+44) (0)1865 275625; (+44) (0)1865 279443; Fax (+44) (0) 1865 275 674; E-mail: michael.mcdonough@chem.ox.ac.uk; christopher.schofield@chem.ox.ac.uk

Supplemental Figures and Legends

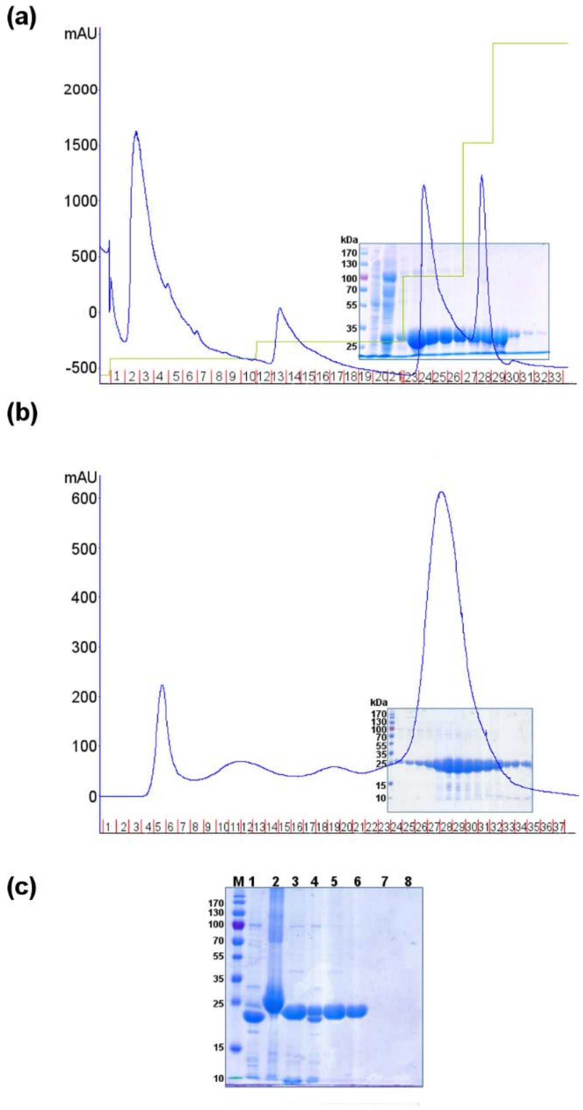

Fig. S1. hETHE1 purification. (a) His-trap purification step: FPLC chromatogram and SDS PAGE of collected fractions. (b) S200 gel filtration purification: FPLC chromatogram and SDS PAGE analysis. (c) SDS PAGE of the *N*-terminal 6X-histidine tag cleavage reaction. The protein tag removal reaction was performed overnight using a 1:50 ratio of 3C protease: recombinant hETHE1 (v/v). Samples: 1. 3C protease; 2. hETHE1 before tag removal; 3. Overnight tag removal reaction mixture; 4. hETHE1 after tag removal with addition of 3C protease 1:1(v/v); 5-6. His-trap column hETHE1 elution after overnight tag removal reaction; 7-8. His-trap column 500 mM imidazole elutions after overnight tag removal reaction.

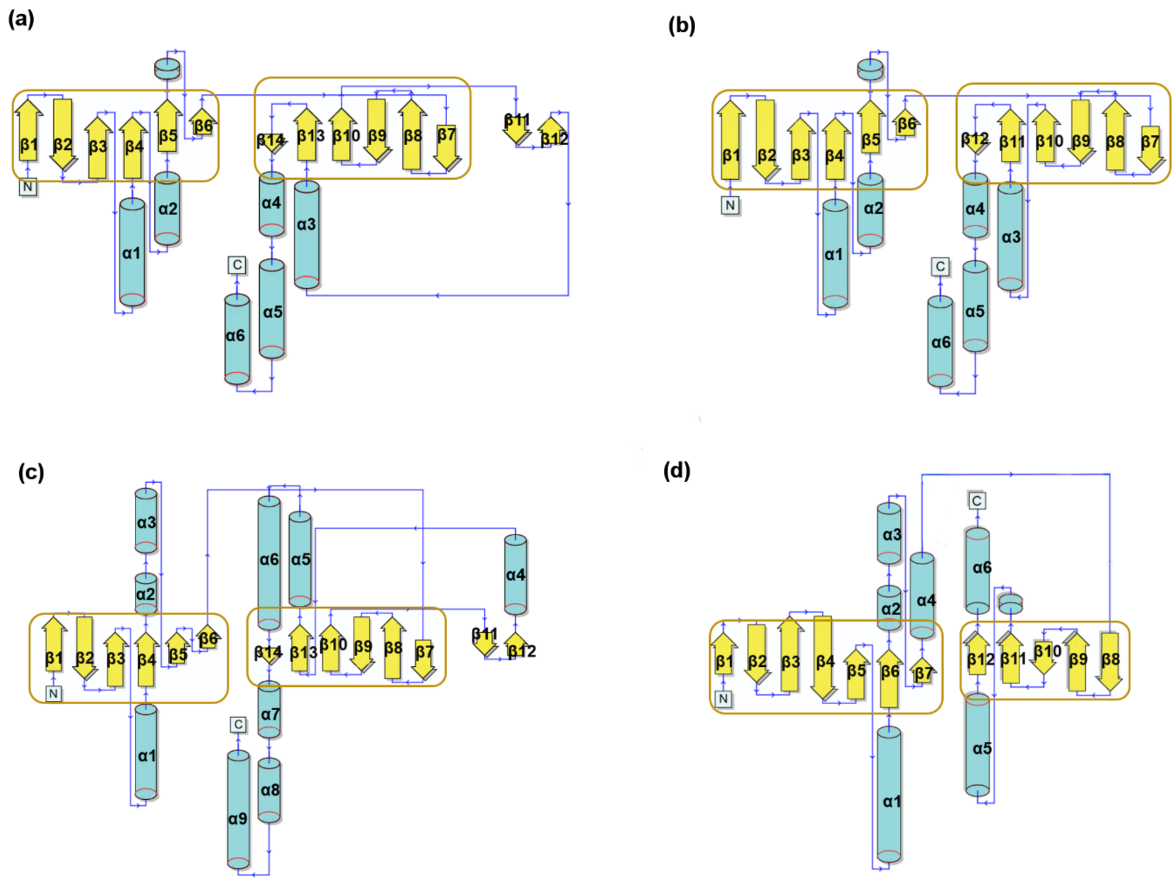

Fig. S2. hETHE1 topology analysis. Topology diagrams of (a) hETHE1; (b) *A. thaliana* ETHE1-like protein; (c) Human glyoxalase II; and (d) a bacterial MBL from *Bacillus cereus* (BcII). β-Sheets I and II are enclosed (yellow line) so they can be distinguished from each other. There is strong structural similarity between ETHE1 from *H. sapiens* and *A. thaliana*; Note the additional β11- β12 hairpin in the region linking β10 and β13 of the core fold of hETHE1 compared to the *A. thaliana* ETHE1 and the resulting different orientation of the loop connecting β12 to α3 and β10 to α3 in *human* and *A.thaliana* ETHE1 respectively. Human glyoxalase II and bacterial BcII show some differences in secondary structure organization compared with the *H. sapiens* and *A. thaliana* ETHE1s. Color coding: α-helices, cyan; β-strands, yellow. The secondary structure elements were defined using PROMOTIF [62] in PDBsum [63].

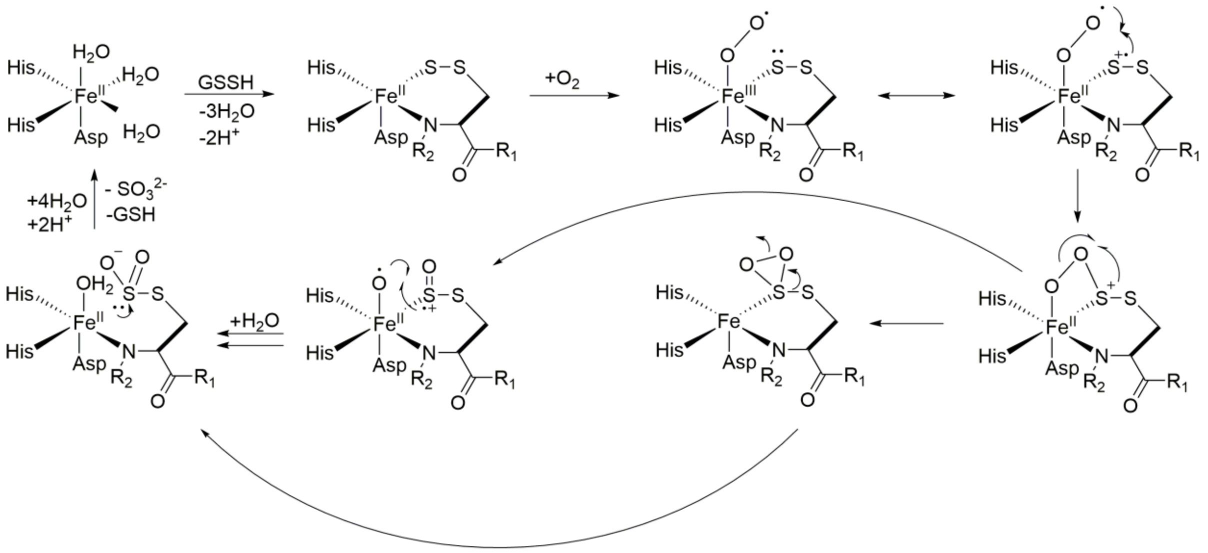

Fig. S3. Possible outline mechanisms for the hETHE1 catalysed reaction (Modification of the existing proposed mechanism [11]). The proposed ETHE1 mechanism is related to that catalyzed by other non-heme iron redox enzymes (i.e. isopenicillin *N* synthase and cysteine dioxygenase) [26-29], and involves initial complexation of the GSSH thiol and GSSH cysteinyl-glycine amide nitrogen to the iron (II), with concomitant displacement of metal bound water molecules, followed by binding of O_2, then oxidation of the ligated sulfur. Note that other mechanisms are possible.
